# Machine learning-based prediction of clinical outcomes and left ventricular ejection fraction improvement after percutaneous coronary intervention in patients with reduced ejection fraction

**DOI:** 10.1093/ehjdh/ztag156

**Published:** 2026-10-27

**Authors:** Lioz Steinbuch, Edward Itelman, Yuval Altman, Bar Yacobi, Osnat Goren-Peyser, Tamir Bental, Yael Shostak, Pablo Codner, Chen Gurevitz, Amos Levi, Guy Witberg, Yeela Talmor Barkan, Ran Kornowski, Leor Perl

**Affiliations:** Department of Medicine B, Rabin Medical Center, Beilinson Campus, Petah-Tikva, and Gray Faculty of Medicine, Tel Aviv University, 39 Jabotinsky Street, Petah Tikva 49100, and 30 Haim Levanon Street, Tel Aviv 6997801, Israel; Department of Cardiology, Rabin Medical Center, Beilinson Campus, Petah-Tikva, and Gray Faculty of Medicine, Tel Aviv University, 39 Jabotinsky Street, Petah Tikva 49100, and 30 Haim Levanon Street, Tel Aviv 6997801, Israel; Department of Cardiology, Rabin Medical Center, Beilinson Campus, Petah-Tikva, and Gray Faculty of Medicine, Tel Aviv University, 39 Jabotinsky Street, Petah Tikva 49100, and 30 Haim Levanon Street, Tel Aviv 6997801, Israel; Beilinson Medical Center Innovation, Artificial Intelligence Center, Rabin Medical Center, Petah-Tikva, Israel; Beilinson Medical Center Innovation, Artificial Intelligence Center, Rabin Medical Center, Petah-Tikva, Israel; Beilinson Medical Center Innovation, Artificial Intelligence Center, Rabin Medical Center, Petah-Tikva, Israel; Beilinson Medical Center Innovation, Artificial Intelligence Center, Rabin Medical Center, Petah-Tikva, Israel; Department of Cardiology, Rabin Medical Center, Beilinson Campus, Petah-Tikva, and Gray Faculty of Medicine, Tel Aviv University, 39 Jabotinsky Street, Petah Tikva 49100, and 30 Haim Levanon Street, Tel Aviv 6997801, Israel; Department of Medicine B, Rabin Medical Center, Beilinson Campus, Petah-Tikva, and Gray Faculty of Medicine, Tel Aviv University, 39 Jabotinsky Street, Petah Tikva 49100, and 30 Haim Levanon Street, Tel Aviv 6997801, Israel; Department of Cardiology, Rabin Medical Center, Beilinson Campus, Petah-Tikva, and Gray Faculty of Medicine, Tel Aviv University, 39 Jabotinsky Street, Petah Tikva 49100, and 30 Haim Levanon Street, Tel Aviv 6997801, Israel; Department of Cardiology, Rabin Medical Center, Beilinson Campus, Petah-Tikva, and Gray Faculty of Medicine, Tel Aviv University, 39 Jabotinsky Street, Petah Tikva 49100, and 30 Haim Levanon Street, Tel Aviv 6997801, Israel; Department of Cardiology, Rabin Medical Center, Beilinson Campus, Petah-Tikva, and Gray Faculty of Medicine, Tel Aviv University, 39 Jabotinsky Street, Petah Tikva 49100, and 30 Haim Levanon Street, Tel Aviv 6997801, Israel; Department of Cardiology, Rabin Medical Center, Beilinson Campus, Petah-Tikva, and Gray Faculty of Medicine, Tel Aviv University, 39 Jabotinsky Street, Petah Tikva 49100, and 30 Haim Levanon Street, Tel Aviv 6997801, Israel; Department of Cardiology, Rabin Medical Center, Beilinson Campus, Petah-Tikva, and Gray Faculty of Medicine, Tel Aviv University, 39 Jabotinsky Street, Petah Tikva 49100, and 30 Haim Levanon Street, Tel Aviv 6997801, Israel; Department of Cardiology, Rabin Medical Center, Beilinson Campus, Petah-Tikva, and Gray Faculty of Medicine, Tel Aviv University, 39 Jabotinsky Street, Petah Tikva 49100, and 30 Haim Levanon Street, Tel Aviv 6997801, Israel; Beilinson Medical Center Innovation, Artificial Intelligence Center, Rabin Medical Center, Petah-Tikva, Israel; Department of Cardiology, Rabin Medical Center, Beilinson Campus, Petah-Tikva, and Gray Faculty of Medicine, Tel Aviv University, 39 Jabotinsky Street, Petah Tikva 49100, and 30 Haim Levanon Street, Tel Aviv 6997801, Israel; Beilinson Medical Center Innovation, Artificial Intelligence Center, Rabin Medical Center, Petah-Tikva, Israel

**Keywords:** Percutaneous coronary intervention, Reduced left ventricular ejection fraction, Machine learning, Risk prediction, Ventricular improvement, Post-PCI prognosis

## Abstract

**Aims:**

The expected clinical and functional trajectory after percutaneous coronary intervention (PCI) in patients with left ventricular dysfunction remains uncertain. Recent trials have highlighted substantial heterogeneity in both clinical outcomes and left ventricular ejection fraction (LVEF) improvement, underscoring the need for improved post-PCI prognostic risk stratification in this high-risk population. This study aimed to develop machine learning models predicting clinical outcomes and LVEF improvement after PCI in patients with reduced LVEF.

**Methods and results:**

This retrospective single-centre study included adults undergoing PCI between 2004 and 2025 with baseline LVEF <45%. Two CatBoost models were developed using 53 routinely available pre- and intra-procedural predictors. The primary endpoint was a 1-year composite of death, myocardial infarction, target vessel revascularization, or coronary artery bypass grafting. The secondary endpoint was an absolute LVEF increase of ≥10 percentage points. Performance was evaluated on a held-out test set using area under the receiver operating characteristic curve (AUC), area under the precision–recall curve (PR-AUC), and enrichment analysis. The dataset included 3336 procedures from 2793 patients (median LVEF 35%). For the primary endpoint (*n* = 2152; event rate 37.5%), the model achieved an AUC of 0.78 (95% CI: 0.73–0.83) and a PR-AUC of 0.70 (95% CI: 0.63–0.77), with event rates of 83.7% in the highest-risk decile and 9.3% in the lowest-risk decile. For LVEF improvement (*n* = 2551; 34.7% improved), performance was moderate (AUC 0.73), with enrichment to 76.9% in the highest-probability decile.

**Conclusion:**

Machine learning models using routinely available patient and procedural data enabled meaningful prognostic stratification in patients with left ventricular dysfunction undergoing PCI, supporting individualized estimation of clinical and functional trajectories after revascularization.

## Graphical Abstract

**Graphical Abstract:**
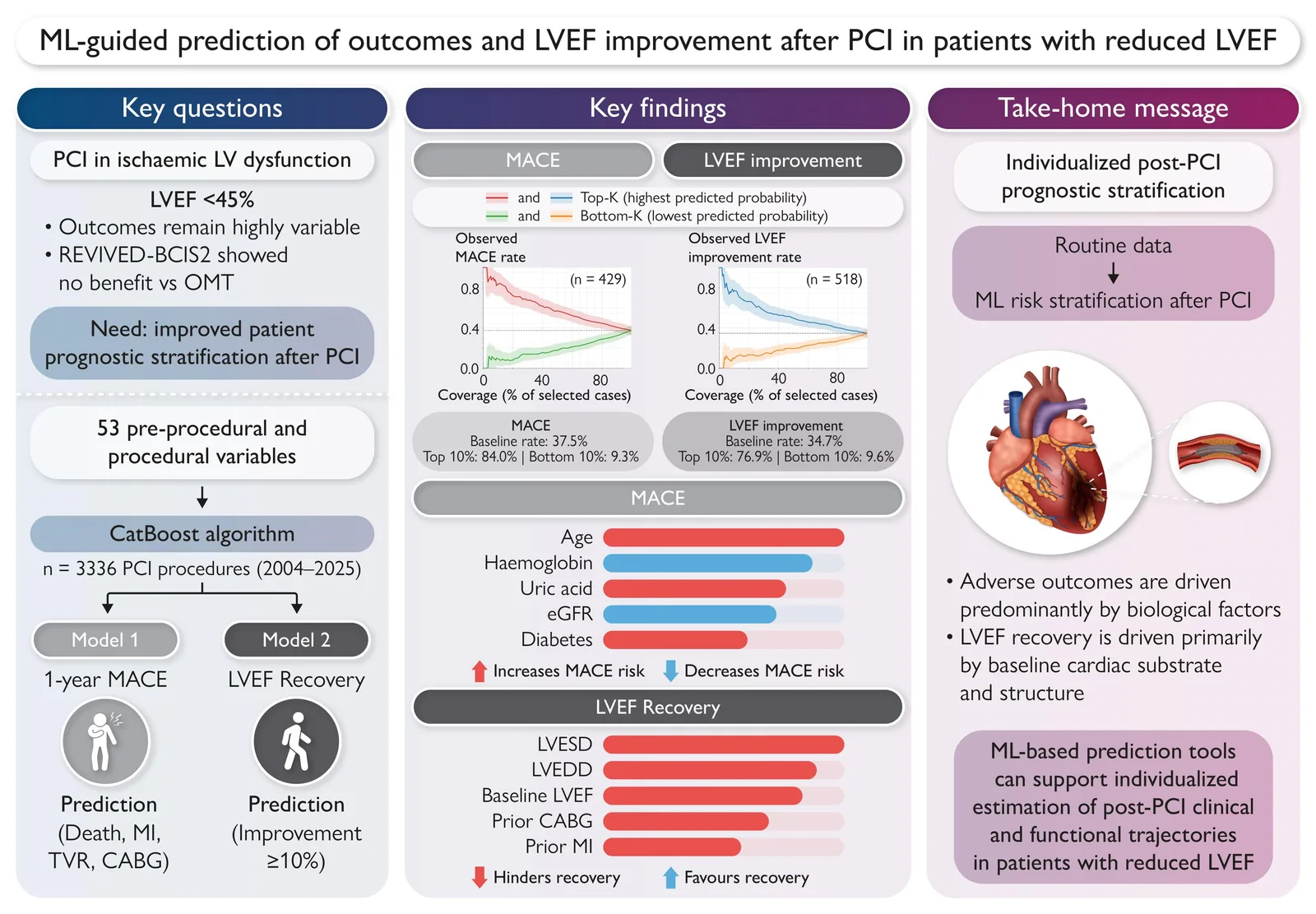

## Introduction

Patients with reduced left ventricular ejection fraction (LVEF) who are considered for percutaneous coronary intervention (PCI) represent one of the most challenging groups in contemporary coronary revascularization. Their outcomes after PCI are highly variable and often difficult to predict. Some patients experience symptomatic improvement, better ischaemic control, and a favourable clinical course, whereas others remain at high risk for death, myocardial infarction (MI), repeat revascularization, or progressive heart failure despite intervention.^1–9^ This heterogeneity reflects the complex interplay of myocardial viability, ischaemic burden, ventricular remodelling, heart failure severity, coronary anatomy, completeness of revascularization, and non-cardiac comorbidity.^2,3,8,9^ The challenge of predicting clinical and echocardiographic outcomes after PCI in this population has become even more relevant in light of recent trial data. In REVIVED-BCIS2, PCI added to contemporary optimal medical therapy did not reduce major adverse clinical outcomes or improve LVEF in patients with ischaemic left ventricular dysfunction, even in the presence of viable myocardium.^9–11^ These findings underscore a central limitation in current practice: the presence of obstructive coronary artery disease and reduced LVEF alone may be insufficient to identify patients most likely to experience favourable clinical and functional trajectories after PCI. More refined tools are therefore needed to support post-PCI prognostic assessment in this high-risk and clinically heterogeneous population.^2,8,9,11^

Prior studies have consistently shown that patients with impaired systolic function have substantially worse outcomes after PCI than those with preserved LVEF, including higher mortality, higher rates of recurrent MI, and greater need for repeat revascularization.^1–5,9^ Traditional risk models, including the Mayo Clinic, CathPCI, New York, and CADILLAC scores, established the prognostic value of age, presentation acuity, clinical comorbidity, procedural factors, and LVEF.^12–15^ More recently, machine learning approaches have shown improved ability to capture non-linear interactions among clinical variables and to predict adverse outcomes after PCI.^16–26^ However, relatively few models have been developed specifically for patients with reduced LVEF as a distinct subgroup, and even fewer have been framed around the practical question clinicians face following revascularization: which patients are likely to follow a favourable course after PCI, and which are likely to remain at high residual risk after it?^26^

Prediction of ventricular improvement represents an additional related challenge in patients with ventricular dysfunction undergoing revascularization. Improvement in LVEF may reflect reperfusion of dysfunctional but viable myocardium and subsequent reverse remodelling and may influence symptoms, heart failure trajectory, timing of reassessment imaging, and downstream device-related decisions. Observational studies suggest that approximately one-half of patients with reduced LVEF may demonstrate some degree of improvement after PCI, although the magnitude and significance of this improvement vary substantially.^27–29^ Recent machine learning studies have also supported the feasibility of predicting LVEF improvement after PCI, demonstrating good predictive performance in selected cohorts.^30^ However, these studies did not specifically focus on the high-risk population of patients with left ventricular dysfunction.

In the present study, we developed two CatBoost-based machine learning models using routinely available pre- and intra-procedural data in patients with baseline LVEF <45% undergoing PCI. The primary model predicts 1-year major adverse cardiovascular events (MACE), defined as a composite of death, MI, target vessel revascularization, or coronary artery bypass grafting, whereas the secondary model predicts post-procedural LVEF improvement. Together, these models are intended to support a more individualized and clinically relevant approach to post-PCI prognostic risk stratification in this high-risk population.

## Methods

### Study design and setting

This retrospective study used routinely collected electronic health record data from Rabin Medical Center (RMC), a tertiary referral centre in Petah Tikva, Israel. The study population included patients undergoing PCI between 2004 and 2025. Each PCI procedure was treated as a separate observation.

### Consent

The institutional review board of RMC approved the study protocol and waived the requirement for individual informed consent due to the retrospective design and use of anonymized data.

### Study population

The study population included adult patients (≥18 years) who underwent PCI at RMC during the study period and had documented baseline LVEF below 45%. Baseline LVEF was obtained from the most recent echocardiographic assessment preceding the index PCI. Follow-up LVEF was obtained from post-PCI echocardiography performed during routine clinical care. The timing of echocardiographic assessments relative to PCI was summarized descriptively.

Each PCI procedure was treated as a separate observation. Because some patients underwent multiple PCI procedures during the study period, patient identifiers were retained to ensure that procedures from the same individual could be handled appropriately during model development and evaluation.

A master analytical dataset was constructed, including all eligible PCI procedures meeting the baseline LVEF criterion for which at least one of the study outcomes (MACE or LVEF improvement) could be determined based on available follow-up data. Cases without a valid label for a specific outcome were excluded from analyses for that endpoint but remained eligible for analysis of the alternate outcome.

### Outcome definitions

#### Clinical outcomes

The primary study outcome was the occurrence of a composite adverse clinical endpoint (MACE), defined as death, MI, target vessel revascularization, or coronary artery bypass grafting.

A case was classified as positive for the MACE outcome if any component of the composite endpoint occurred within 365 days after the index PCI procedure. Cases were classified as negative if no component of the composite endpoint occurred, and follow-up duration exceeded 365 days after the procedure.

#### LVEF improvement

The secondary study outcome was improvement in LVEF following PCI. It was defined based on the change between baseline and follow-up echocardiographic measurements. Left ventricular ejection fraction was assessed by transthoracic echocardiography using the Simpson biplane method when available and by visual estimation otherwise. Baseline LVEF was obtained from the echocardiographic examination preceding the index PCI procedure, and follow-up LVEF values were obtained from subsequent echocardiographic studies performed after the procedure.

Left ventricular ejection fraction improvement was defined as an increase of ≥10 percentage points between the baseline and follow-up measurements. This threshold was selected to represent a clinically meaningful change in ventricular systolic function while exceeding the expected variability of routine echocardiographic LVEF assessment.^31, 32^ Cases with a change in LVEF of ≤0 percentage points were classified as not demonstrating improvement. To create clearly separated outcome groups, procedures with intermediate changes in LVEF (>0 and <10%) were excluded from analyses of this endpoint.

### Model development

The master analytical dataset was partitioned at the patient level into a development set (80%) and a held-out test set (20%), ensuring that all procedures from a given patient were assigned to a single split to prevent information leakage. Stratification was performed separately for each outcome to preserve outcome prevalence. Because outcome availability differed across endpoints, this process yielded two partially overlapping analysis datasets, one for each prediction task.

Predictive models were developed using gradient boosting decision trees implemented with the CatBoost framework. Predictor variables included demographic characteristics, clinical history and comorbidities, laboratory measurements, echocardiographic parameters, as well as procedural and angiographic characteristics recorded during the index PCI. Percutaneous coronary intervention completeness was calculated as the ratio of coronary territories treated during the index procedure to the number of diseased vessels supplying these territories and was categorized as higher (>0.5) or lower (≤0.5). The three territories were defined according to the typical anatomical distribution of the major epicardial arteries (left anterior descending, left circumflex, and right coronary arteries): the anterior/septal, lateral/posterolateral, and inferior/right territories, respectively. The intended prediction time was the completion of the index PCI, at which point the clinical, angiographic, and procedural inputs required by the model were available. The final modelling dataset included 53 predictors (see Supplementary material online, *Table S1*).

Two separate models were trained using the same predictor set: one to predict the MACE composite outcome and one to predict improvement in LVEF, as defined above. Model development was performed using the development dataset. Hyper-parameters were selected using cross-validated grid search within the development set. With the selected configuration fixed, 10-fold cross-validation was used to generate out-of-fold predictions for subsequent calibration and operating-threshold selection. The final model for each outcome was trained on the entire development dataset using the selected model configuration. The CatBoost models were trained using 1500 boosting iterations with a tree depth of 6, a learning rate of 0.05, and L2 regularization of 3. Class imbalance was addressed using balanced class weights. Binary variables were coded as 0 or 1, with missing values retained as missing. No feature scaling or imputation was applied, and missing values were handled natively by the CatBoost algorithm. The held-out test set was not used for hyper-parameter selection, calibration fitting, or operating-threshold selection and was reserved for final model evaluation.

### Model evaluation

Model performance was assessed using the held-out test set based on calibrated predicted probabilities. Discrimination was evaluated using the area under the receiver operating characteristic curve (AUC) and the area under the precision–recall curve (PR-AUC). Additional classification metrics included accuracy, sensitivity, specificity, positive predictive value (PPV), negative predictive value (NPV), F1 score, and balanced accuracy.

Confidence intervals for test-set performance metrics were estimated using bootstrap resampling with 1000 iterations.

To illustrate performance across clinically relevant operating points, classification metrics were reported at three probability thresholds. A balanced threshold was selected to maximize balanced accuracy, and a screening threshold was selected to maximize PPV while maintaining sensitivity ≥0.8. Thresholds were determined using out-of-fold predictions generated during cross-validation within the development dataset. For reference, performance at the default probability threshold of 0.5 was also reported.

Probability calibration was performed using Platt scaling. A logistic regression calibration model was fitted using out-of-fold predicted probabilities from the development dataset and the corresponding observed outcomes. The resulting calibration model was then applied to predicted probabilities generated by the final models on the test dataset.

The association between observed LVEF improvement and 1-year MACE was evaluated among cases with both endpoint labels available using Fisher’s exact test.

### Model interpretability

Model interpretability was assessed using SHapley Additive exPlanations (SHAP). SHAP values were used to quantify the direction and magnitude of each predictor’s contribution to the model’s output. Global feature importance was summarized using the mean absolute SHAP value for each predictor across the held-out test set.

### Enrichment analysis

To evaluate the models’ ability to stratify cases by outcome risk, enrichment analysis was performed on the held-out test dataset. Cases were ranked according to predicted probability. For predefined coverage levels corresponding to the top 5, 10, 20, and 30% of predictions, outcome rates within the high-risk subsets were compared with those among the remaining cases using Fisher’s exact test. Odds ratios and corresponding *P*-values were calculated to quantify enrichment of outcomes among cases with the highest predicted probabilities. Complementary analyses were also performed for subsets corresponding to the lowest predicted probabilities (bottom 5, 10, 20, and 30%) to assess outcome depletion in low-probability subsets.

In addition, cumulative outcome rates were evaluated across a range of coverage levels to characterize enrichment as a continuous function of model confidence. Cumulative outcome rates were evaluated for both the highest- and lowest-probability subsets. Confidence intervals for these enrichment curves were estimated using bootstrap resampling (2000 iterations). An exploratory joint stratification analysis was performed by identifying cases with both low predicted clinical risk and high predicted probability of LVEF improvement and descriptively comparing their baseline characteristics with those of the remaining cases. Model performance was also evaluated in the subgroup of held-out test cases with baseline LVEF ≤40%.

A TRIPOD + AI checklist is provided in Supplementary material online, *Table S2*.

## Results

### Study cohort

Among 35 902 PCI procedures in the full cohort, 3336 were included in the final analysis, representing 2793 patients with baseline LVEF < 45% treated at RMC between 2004 and 2025 (*Figure 1*). The dataset was divided at the patient level into a development set (2669 procedures from 2234 patients) and a held-out test set (667 procedures from 559 patients). Baseline characteristics are presented in *Table 1*, and discharge cardiovascular and heart failure-related pharmacotherapy is summarized in Supplementary material online, *Table S4*.

**Figure 1:**
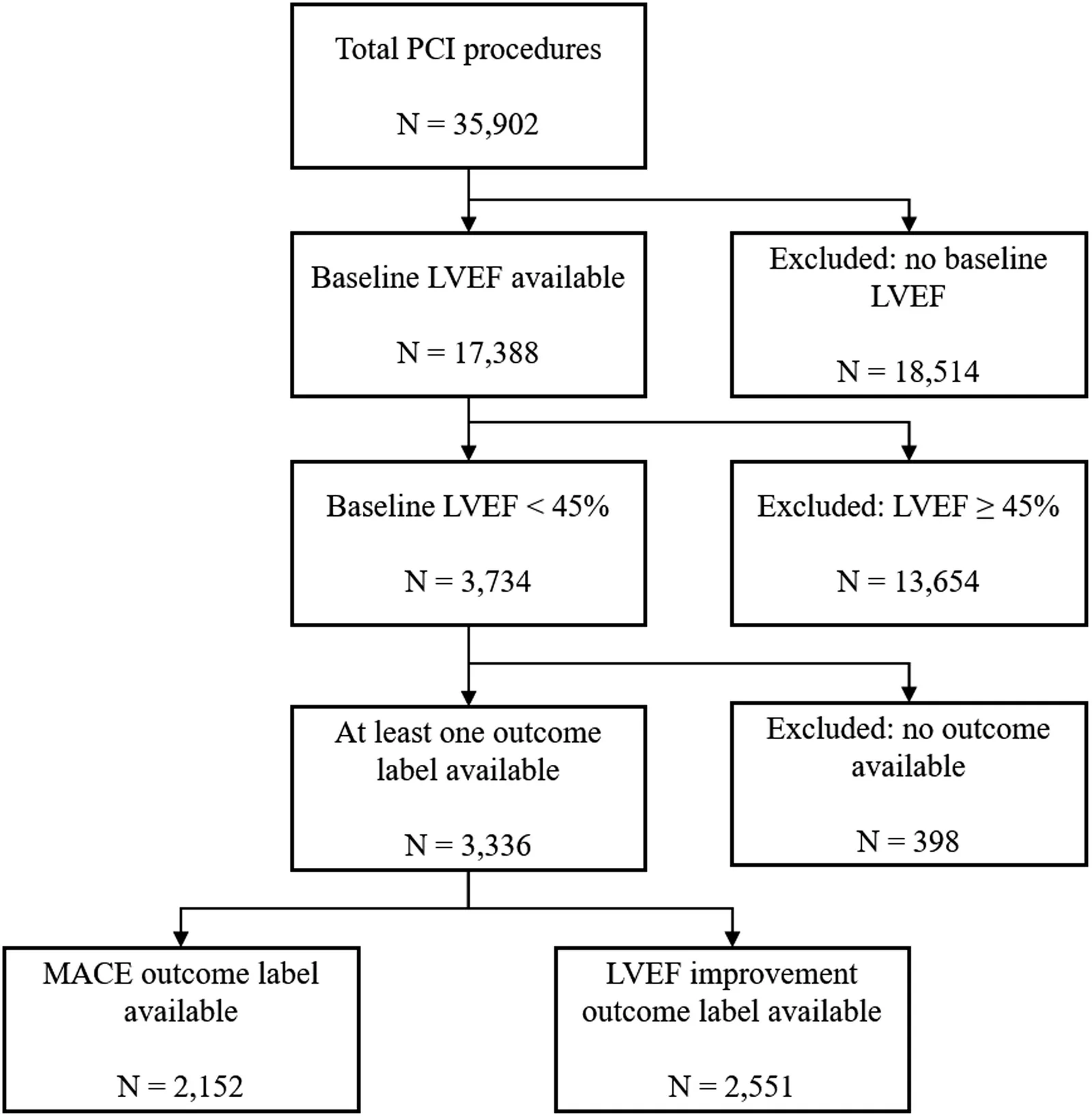
Flow diagram of study cohort selection and dataset construction. Flow diagram illustrating the selection of the study cohort from all percutaneous coronary intervention procedures performed during the study period. Procedures were sequentially filtered based on availability of baseline left ventricular ejection fraction, inclusion of cases with left ventricular ejection fraction <45%, and availability of at least one outcome label. The final analytical dataset was used to derive two partially overlapping cohorts for prediction of the composite clinical outcome (MACE: death, myocardial infarction, target vessel revascularization, or coronary artery bypass grafting) and left ventricular ejection fraction improvement.

**Table 1:** Baseline characteristics of the study cohort

Variable	Overall cohort	Development set	Test set
**Demographics**			
Age (years)	69.00 [61.00, 77.00]	69.00 [61.00, 77.00]	69.00 [62.00, 77.00]
Female sex	668 (20.0%)	537 (20.1%)	131 (19.6%)
**Clinical history**			
Diabetes mellitus	2057 (61.7%)	1659 (62.2%)	398 (59.7%)
Hypertension	2769 (83.0%)	2223 (83.3%)	546 (81.9%)
Smoking history	1157 (34.7%)	906 (33.9%)	251 (37.6%)
Myocardial infarction	1209 (36.2%)	958 (35.9%)	251 (37.6%)
Chronic obstructive pulmonary disease	409 (12.3%)	317 (11.9%)	92 (13.8%)
Peripheral vascular disease	201 (6.0%)	164 (6.1%)	37 (5.5%)
Atrial fibrillation	395 (11.8%)	328 (12.3%)	67 (10.0%)
Malignancy	467 (14.0%)	372 (13.9%)	95 (14.2%)
Stroke	363 (10.9%)	290 (10.9%)	73 (10.9%)
Dementia	102 (3.1%)	78 (2.9%)	24 (3.6%)
Congestive heart failure	594 (17.8%)	478 (17.9%)	116 (17.4%)
CABG	711 (21.3%)	581 (21.8%)	130 (19.5%)
**Laboratory values**			
Haemoglobin (g/dL)	11.30 [9.40, 12.90]	11.20 [9.38, 12.90]	11.50 [9.40, 12.90]
Platelet count (×10^9^/L)	214.00 [174.00, 268.00]	213.00 [174.00, 269.00]	215.50 [175.00, 264.00]
Fibrinogen (mg/dL)	570.00 [450.00, 721.25]	572.00 [452.25, 722.00]	563.50 [444.50, 718.00]
HDL (mg/dL)	34.00 [28.00, 42.00]	34.00 [28.00, 42.00]	33.00 [27.00, 41.00]
LDL (mg/dL)	86.00 [69.00, 99.00]	86.00 [69.00, 99.00]	85.00 [67.00, 99.00]
Triglycerides (mg/dL)	154.00 [107.00, 227.00]	154.00 [108.00, 228.00]	151.50 [107.00, 221.00]
MDRD eGFR (mL/min/1.73 m^2^)	71.44 [49.67, 91.27]	71.42 [48.99, 91.49]	71.63 [52.79, 89.61]
**Echocardiographic parameters**			
Left ventricular ejection fraction (%)	35.00 [30.00, 40.00]	35.00 [30.00, 40.00]	35.00 [30.00, 40.00]
Left ventricular end-diastolic diameter (mm)	53.00 [48.00, 58.00]	53.00 [48.00, 58.00]	53.00 [48.00, 57.00]
Left ventricular end-systolic diameter (mm)	40.00 [35.00, 47.00]	41.00 [35.00, 47.00]	40.00 [34.00, 47.00]
Left ventricular posterior wall thickness (mm)	10.00 [9.00, 11.00]	10.00 [9.00, 11.00]	10.00 [9.00, 11.00]
Aortic stenosis (≥mild)	255 (10.6%)	203 (10.6%)	52 (10.6%)
Mitral regurgitation (≥mild)	1779 (73.8%)	1435 (74.9%)	344 (69.6%)
Tricuspid regurgitation (≥mild)	1112 (46.4%)	887 (46.5%)	225 (46.0%)
**Procedural context**			
PCI count (including index procedure)	1.00 [1.00, 2.00]	1.00 [1.00, 2.00]	1.00 [1.00, 2.00]
Cardiogenic shock at presentation	66 (2.0%)	56 (2.1%)	10 (1.5%)
PCI completeness index >0.5	1190 (35.9%)	959 (36.2%)	231 (34.8%)
Proximal major vessel treated	1664 (49.9%)	1350 (50.6%)	314 (47.1%)

Continuous variables are presented as median (interquartile range), and categorical variables as number (percentage). Values are calculated based on available data for each variable. Valve disease variables are presented as the presence of at least mild disease (grade ≥2). Development and test sets were defined using a patient-level split of the full cohort.

Because outcome labels were not available for both endpoints in all procedures, the effective sample size differed between the two prediction tasks. For the primary outcome, the dataset included 2152 procedures, with a composite event rate of 37.5%. For the secondary endpoint, the dataset included 2551 procedures, of which 34.7% met the improvement criterion.

Echocardiographic timing data were available for all relevant cases. In the full analytical cohort, the median interval from baseline echocardiography to PCI was 24 days [interquartile range (IQR) 3–116 days]. Among cases included in the LVEF improvement endpoint, the median interval from PCI to follow-up echocardiography was 85 days (IQR 3–342 days).

### Prediction of clinical outcomes

The clinical model demonstrated good discrimination on the held-out test dataset, with an AUC of 0.78 (95% CI 0.73–0.83) and a PR-AUC of 0.70 (95% CI 0.63–0.77). Classification performance is summarized in *Table 2*. Enrichment analysis demonstrated a substantial depletion of event rates among cases with the lowest predicted probabilities. The MACE rate was 9.3% among the bottom 10% of predictions, compared with 40.7% among the remaining cases. Conversely, higher event rates were observed among cases with the highest predicted probabilities, with the MACE rates reaching 83.7% among the top 10% of predictions compared with 32.4% among the remaining cases. Significant enrichment persisted across broader coverage levels (*Table 3*, *Figure 2*).

**Figure 2:**
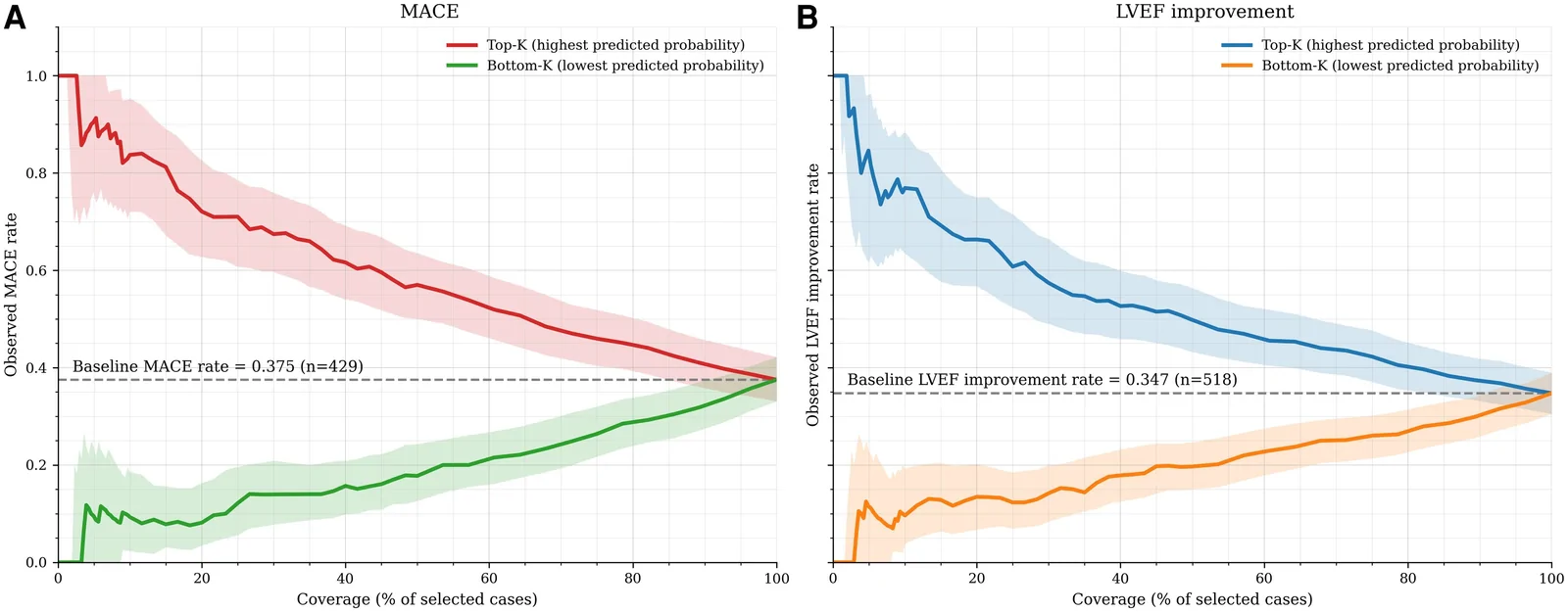
Outcome rates across increasing coverage of predicted probability for the MACE and left ventricular ejection fraction improvement models. Outcome rates across increasing coverage levels of predicted probability on the held-out test dataset for the MACE (*A*) and left ventricular ejection fraction improvement (*B*) models. Cases were ranked according to predicted probability, and cumulative outcome rates were calculated across a range of coverage levels for both the highest (top-K) and lowest (bottom-K) predicted probabilities. Shaded areas indicate 95% confidence intervals. The dashed horizontal line represents the overall outcome rate in the corresponding test dataset. Top-K curves represent outcome concentration among higher-risk predictions, whereas bottom-K curves represent outcome depletion among lower-risk predictions.

**Table 2:** Model performance on the test dataset

Model	Operating point	Accuracy	Sensitivity	Specificity	PPV	NPV	F1 score	Balanced accuracy
MACE	Balanced	0.70 (0.66–0.74)	0.71 (0.64–0.79)	0.69 (0.63–0.74)	0.58 (0.51–0.65)	0.80 (0.75–0.85)	0.64 (0.58–0.69)	0.70 (0.66–0.75)
	Screening	0.68 (0.64–0.73)	0.79 (0.73–0.85)	0.62 (0.56–0.68)	0.55 (0.50–0.62)	0.83 (0.78–0.88)	0.65 (0.60–0.70)	0.70 (0.66–0.75)
	Default	0.74 (0.70–0.78)	0.57 (0.50–0.65)	0.84 (0.79–0.88)	0.68 (0.59–0.75)	0.76 (0.72–0.81)	0.62 (0.55–0.68)	0.70 (0.66–0.75)
LVEF improvement	Balanced	0.67 (0.63–0.71)	0.64 (0.57–0.71)	0.68 (0.63–0.73)	0.52 (0.45–0.59)	0.78 (0.73–0.82)	0.57 (0.52–0.63)	0.66 (0.62–0.70)
	Screening	0.60 (0.55–0.64)	0.82 (0.76–0.87)	0.48 (0.43–0.53)	0.46 (0.40–0.51)	0.84 (0.78–0.88)	0.59 (0.53–0.64)	0.65 (0.61–0.69)
	Default	0.71 (0.68–0.75)	0.43 (0.36–0.50)	0.87 (0.83–0.90)	0.63 (0.54–0.71)	0.74 (0.70–0.78)	0.51 (0.44–0.58)	0.65 (0.61–0.69)

Performance is reported for three operating thresholds: balanced, optimizing balanced accuracy; screening, maximizing positive predictive value under the constraint of sensitivity ≥0.80; and default (probability threshold 0.5). Values are presented as point estimates with 95% confidence intervals derived from bootstrap resampling (*n* = 1000).

**Table 3:** Outcome enrichment and depletion across selected prediction strata on the test dataset

Outcome	Subset	Coverage	Cases (*n*)	Outcome rate (subset)	Outcome rate (remaining)	Odds ratio	*P*-value
MACE	Bottom	5%	21	9.5%	39.0%	0.16	5.2 × 10^−3^
	Bottom	10%	43	9.3%	40.7%	0.15	2.1 × 10^−5^
	Bottom	20%	86	8.1%	44.9%	0.11	2.1 × 10^−11^
	Bottom	30%	129	14.0%	47.7%	0.18	6.8 × 10^−12^
	Top	5%	21	90.5%	34.8%	17.80	3.8 × 10^−7^
	Top	10%	43	83.7%	32.4%	10.74	7.4 × 10^−11^
	Top	20%	86	72.1%	28.9%	6.37	3.9 × 10^−13^
	Top	30%	129	67.4%	24.7%	6.33	1.2 × 10^−16^
LVEF improvement	Bottom	5%	26	11.5%	36.0%	0.23	1.0 × 10^−2^
	Bottom	10%	52	9.6%	37.6%	0.18	2.2 × 10^−5^
	Bottom	20%	104	13.5%	40.1%	0.23	1.3 × 10^−7^
	Bottom	30%	155	14.2%	43.5%	0.21	2.9 × 10^−11^
	Top	5%	26	84.6%	32.1%	11.63	1.1 × 10^−7^
	Top	10%	52	76.9%	30.0%	7.76	1.2 × 10^−10^
	Top	20%	104	66.3%	26.8%	5.38	1.6 × 10^−13^
	Top	30%	155	57.4%	25.1%	4.03	3.5 × 10^−12^

Cases were ranked according to predicted probability on the held-out test dataset. Outcome rates were calculated for predefined coverage levels corresponding to both the highest (top-K) and lowest (bottom-K) predicted probabilities and compared with the remaining cases using Fisher’s exact test. Odds ratios >1 indicate enrichment, and odds ratios <1 indicate depletion of the outcome in the selected subset.

Model interpretability analysis indicated that the most influential predictors primarily reflected baseline clinical risk and systemic health status, including demographic factors (age), laboratory markers (haemoglobin, uric acid, MDRD eGFR, and platelet count), comorbidity burden (diabetes mellitus), and echocardiographic measures, such as left ventricular dysfunction and aortic stenosis (*Figure 3*).

**Figure 3:**
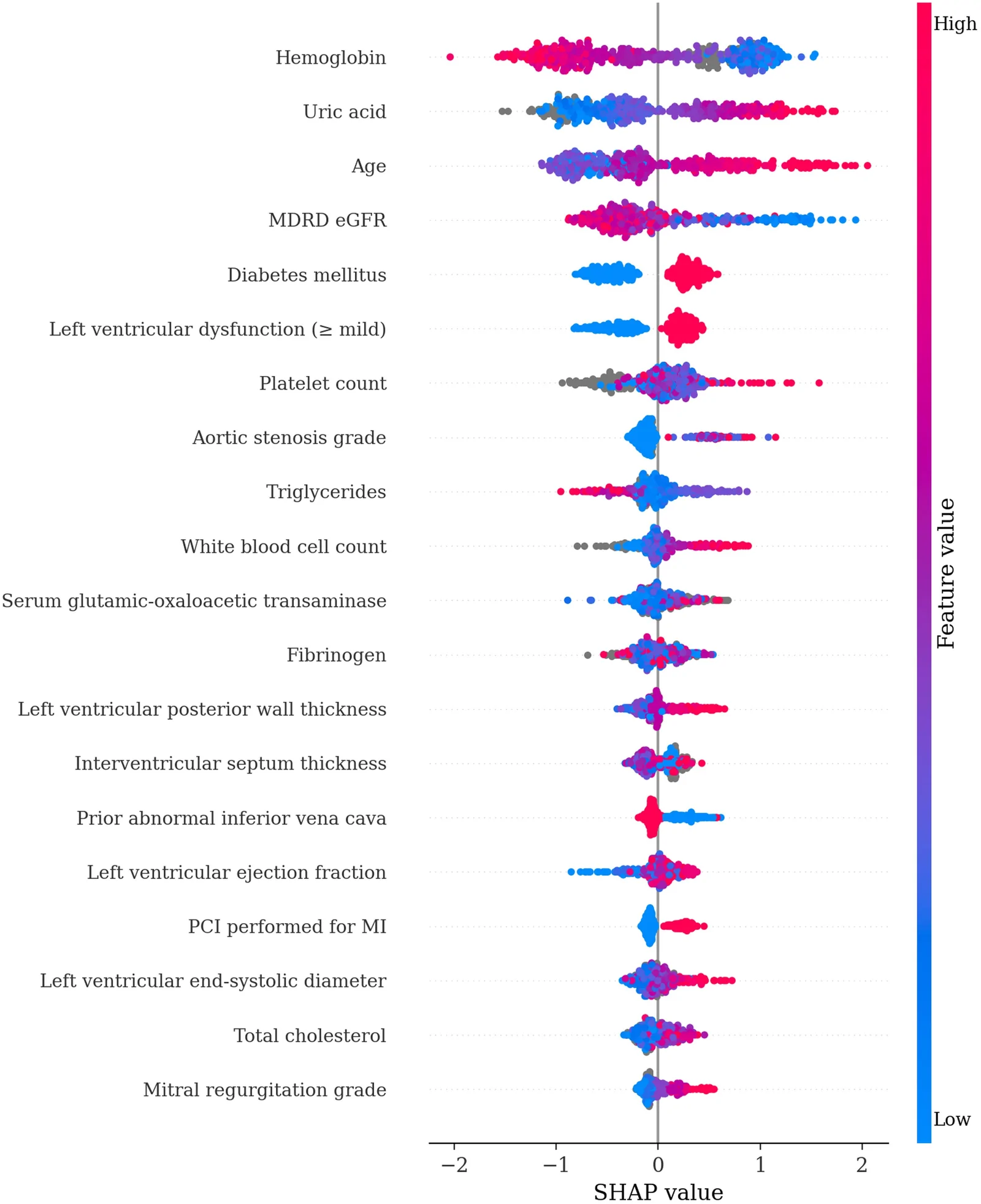
SHAP summary plot for the MACE model. SHapley Additive exPlanations (SHAP) summary plot illustrating the relative importance and direction of effect of individual predictors on model output for the composite clinical outcome (MACE: death, myocardial infarction, target vessel revascularization, or coronary artery bypass grafting). Each point represents a single observation, with colour indicating the feature value. Features are ordered by mean absolute SHAP value. Only the top 20 predictors are shown.

### Prediction of LVEF improvement

The LVEF improvement model demonstrated moderate discrimination on the held-out test dataset, with an AUC of 0.73 (95% CI 0.68–0.77) and a PR-AUC of 0.62 (95% CI 0.54–0.69). Classification performance across selected operating thresholds is presented in *Table 2*. Enrichment analysis demonstrated a substantial increase in improvement rates among cases with the highest predicted probabilities. The improvement rate reached 76.9% among the top 10% of predictions compared with 30.0% among the remaining cases. Conversely, lower improvement rates were observed among cases with the lowest predicted probabilities, indicating depletion of improvement in low-probability subsets. Significant enrichment remained evident across broader coverage levels (*Table 3*, *Figure 2*). Calibration curves for both models are presented in Supplementary material online, *Figure S1*.

Model interpretability analysis indicated that the most influential predictors were primarily related to baseline ventricular structure and systolic function, including left ventricular end-systolic and end-diastolic diameters and baseline LVEF, along with clinical factors such as prior CABG, congestive heart failure, MI, age, and renal/metabolic markers, including MDRD eGFR and uric acid (*Figure 4*).

**Figure 4:**
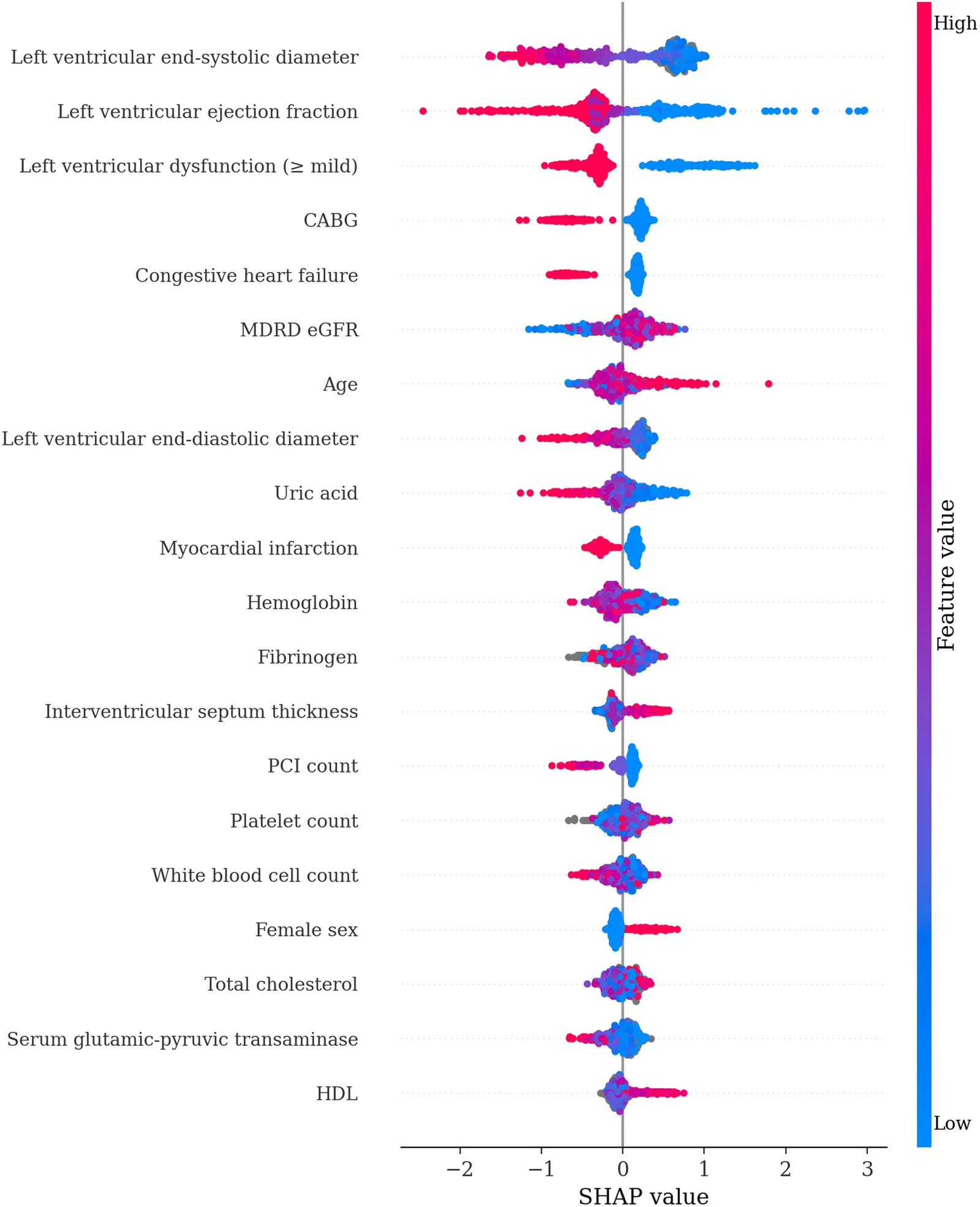
SHapley Additive exPlanations (SHAP) summary plot for the left ventricular ejection fraction improvement model. SHAP summary plot illustrating the relative importance and direction of effect of individual predictors on model output for left ventricular ejection fraction improvement. Each point represents a single observation, with colour indicating the feature value. Features are ordered by mean absolute SHAP value. Only the top 20 predictors are shown.

### Model performance in patients with baseline LVEF ≤40%

Among held-out test cases with baseline LVEF ≤40%, model performance was similar to the primary analysis for both MACE [AUC 0.78 (95% CI 0.73–0.82), PR-AUC 0.70 (95% CI 0.63–0.77)] and LVEF improvement [AUC 0.73 (95% CI 0.69–0.78), PR-AUC 0.62 (95% CI 0.55–0.69)].

### Exploratory joint stratification analysis

To further characterize patients with favourable predicted outcomes, we examined the subset of cases with both low predicted MACE risk (bottom 25% of predictions) and high predicted probability of LVEF improvement (top 25% of predictions). This subgroup comprised 23 of 280 common test cases (8.2%). Within this subgroup, the observed MACE rate was 13.0%, compared with 40.1% in the remaining cases, while the rate of LVEF improvement was 65.2 vs. 32.7%, respectively. Baseline characteristics of this subgroup, compared with the remaining cases, are presented in Supplementary material online, *Table S3*. Descriptively, this subgroup was characterized by lower prevalence of diabetes mellitus and prior MI, higher haemoglobin levels, better renal function, and less advanced ventricular structural abnormalities.

#### Association between LVEF improvement and MACE

Among 1367 cases with both endpoint labels available, observed LVEF improvement was associated with a lower MACE rate compared with no LVEF improvement (27.0 vs. 41.5%; OR 0.52, *P* < 0.001).

## Discussion

In this retrospective cohort of patients with baseline LVEF < 45% undergoing PCI, we developed and evaluated two machine learning models addressing complementary clinical questions: the risk of adverse clinical outcomes during the first year after PCI and the probability of improvement in ventricular systolic function after revascularization. For the clinical outcome model, event rates reached 83.7% among the top 10% of predicted cases vs. 32.4% among the remaining patients, indicating marked enrichment of adverse outcomes in a high-risk subgroup. Conversely, the bottom 10% of predicted cases had a MACE rate of only 9.3%, compared with 40.7% among the remaining cases. Patients with reduced LVEF are broadly considered high risk, yet their likelihood of adverse events after revascularization varies substantially.^2,8,9,16–26,33–35^ The present model may therefore help identify patients who warrant closer follow-up or intensified adjunctive therapy, while also identifying lower-risk patients with a more favourable predicted post-PCI trajectory.

The LVEF improvement model provided complementary information, identifying a top decile in which 76.9% of patients met the improvement definition, compared with 30.0% in the remainder. In patients with ischaemic left ventricular dysfunction, estimating the likelihood of subsequent improvement in systolic function after PCI is clinically relevant because it informs symptom expectations, heart failure management, follow-up imaging, and downstream device-related decisions. However, randomized evidence has raised doubts about the causal effect of PCI on improvement in chronic ischaemic ventricular dysfunction. In REVIVED-BCIS2, PCI did not improve LVEF or clinical outcomes beyond optimal medical therapy despite viability-based selection.^9,10^ Our findings therefore suggest that patients more or less likely to experience measurable improvement in LVEF after PCI may be identifiable, although such improvement should not be interpreted as being attributable solely to PCI.

Taken together, the two models support a more personalized approach to post-PCI prognostic assessment among patients selected for revascularization. A patient with a high predicted probability of LVEF improvement and a low predicted risk of adverse events may be more likely to follow a favourable post-PCI clinical and functional trajectory, whereas a patient with a low predicted probability of ventricular improvement and a high predicted clinical risk may be more likely to experience an unfavourable post-PCI course and may warrant closer follow-up and intensified clinical management. This concept was further supported by the exploratory analysis of the subgroup with both low predicted MACE risk and high predicted probability of LVEF improvement. Although this subgroup represented only 8.2% of common test cases, it demonstrated a favourable profile, with lower observed MACE rates (13.0 vs. 40.1%) and higher rates of LVEF improvement (65.2 vs. 32.7%) than the remaining cases. In addition, observed LVEF improvement was associated with lower 1-year MACE rates among cases with both endpoint labels available, supporting the clinical relevance of ventricular functional improvement as a secondary endpoint while not establishing a causal relationship between improvement and clinical outcomes.

The interpretability analyses provided additional biological insight into the two models. The highest-ranking predictors of adverse clinical outcomes were predominantly systemic rather than coronary-specific variables, with high-ranking predictors including haemoglobin, age, uric acid, and renal function indices, suggesting that post-PCI risk in patients with reduced LVEF is driven largely by physiological reserve and cardio–renal–metabolic vulnerability. In contrast, the highest-ranking predictors of LVEF improvement were more strongly related to baseline ventricular structure and systolic function, with less prominent contribution from systemic or metabolic variables. Notably, lower baseline LVEF showed a positive contribution to predicted improvement, which may reflect greater potential for measurable improvement among patients starting from more severely reduced systolic function. This divergence supports the concept that ventricular improvement after PCI is shaped primarily by myocardial substrate and remodelling potential, whereas adverse outcomes are influenced more strongly by global biological vulnerability. Although these patterns are broadly consistent with clinical intuition, their independent emergence from interpretable machine learning models provides novel empirical support for these pathophysiological distinctions and reinforces the plausibility of the models as post-PCI prognostic tools.

Compared with prior machine learning models for prediction of outcomes after PCI, the present study offers several advances. Its novelty lies not in identifying previously unrecognized individual predictors, but in integrating routinely available demographic, clinical, laboratory, echocardiographic, procedural, and angiographic variables into a clinically interpretable framework for individualized post-PCI prognostic assessment. It focuses specifically on patients with reduced LVEF, a high-risk population with substantial heterogeneity in outcomes after PCI; pairs prediction of adverse clinical outcomes with prediction of ventricular functional improvement; and identifies both high-risk and low-risk post-PCI phenotypes. Taken together, these features extend the role of machine learning in this setting towards individualized estimation of post-PCI clinical and functional trajectory in a high-risk population undergoing PCI.

Because both models use routinely available pre- and intra-procedural variables, they may be suitable for integration into the electronic health record as point-of-care prognostic tools. Once diagnostic angiography is completed and the intended PCI strategy is defined, automated extraction of these data could enable real-time generation of individualized estimates of the expected post-PCI clinical and functional trajectory during the index procedure itself. These estimates could support multidisciplinary ‘heart team’ discussions, post-PCI prognostic counselling, and risk-adapted planning of follow-up intensity and adjunctive clinical management.

Several limitations should be acknowledged. This was a retrospective single-centre study, and external validation is necessary before clinical adoption. Prospective validation in real-world clinical workflows will be essential to confirm model performance, assess generalizability, and define the practical role of these tools in post-PCI prognostic risk stratification. A logical next step would be prospective validation in a consecutive real-world cohort of patients with reduced LVEF undergoing PCI, with model predictions generated after completion of the procedure during the same hospitalization but initially withheld from treating clinicians. Such a design would allow assessment of discrimination, calibration, subgroup performance, and clinical usability under routine workflow conditions before subsequent evaluation of the model’s impact on post-PCI management and follow-up planning. The study period spanned more than two decades, during which clinical practice, device technology, and medical therapy evolved; potential temporal effects on model performance were not explicitly evaluated. The observational design precludes causal inference regarding the effect of PCI on either ventricular improvement or clinical events and does not permit direct estimation of treatment benefit relative to alternative strategies. Because all patients in the analytical cohort underwent PCI, the models cannot identify patients who would benefit from PCI compared with conservative management or guide PCI vs. non-PCI treatment selection. Similarly, LVEF improvement should be interpreted as post-PCI functional change rather than improvement attributable solely to PCI. Post-procedural medical therapy may also have influenced both clinical outcomes and LVEF improvement; although discharge medication data were available descriptively, longitudinal treatment patterns, dose intensity, and adherence were not explicitly modelled in the present analysis. In addition, the timing of baseline and follow-up echocardiographic assessments was not standardized across the cohort, reflecting the real-world nature of the study. Variation in the interval between PCI and post-procedural LVEF reassessment may therefore have influenced the observed magnitude of ventricular improvement and allowed for interval changes related to other factors. Exclusion of intermediate cases from the LVEF improvement endpoint may induce selection bias and limit applicability to patients with more modest improvement. In addition, because inclusion required documented baseline echocardiographic assessment, patients undergoing urgent PCI without formal pre-procedural LVEF evaluation may have been excluded, potentially introducing selection bias and limiting generalizability to the highest-risk urgent PCI populations. Although the primary analysis used an LVEF threshold of <45% to capture a broader population with clinically relevant systolic dysfunction, model performance was similar in the subgroup with baseline LVEF ≤40%, supporting the robustness of the findings within the more conventionally defined HFrEF range. The models incorporated routinely available clinical, laboratory, echocardiographic, procedural, and angiographic variables, including completeness of revascularization, proximal major-vessel treatment, and the treated coronary territory. However, detailed lesion-level anatomy and measures of procedural complexity and success—including final residual stenosis and post-procedural coronary flow—were not consistently available and therefore could not be incorporated. These factors may provide additional prognostic information. The study also did not incorporate certain clinically relevant variables, including natriuretic peptide levels (BNP/NT-proBNP), which were not consistently available across the cohort, or gold-standard imaging measures of myocardial injury or ischaemia, such as late gadolinium enhancement or stress-perfusion cardiac magnetic resonance. Instead, the study relied on LVEF change as a surrogate of functional improvement, which may not fully reflect the underlying myocardial substrate. Finally, the clinical endpoint combines outcomes of differing clinical weight, although it was selected to reflect a broad spectrum of clinically relevant adverse events during the first year after PCI in a high-risk population.

In conclusion, among patients with reduced LVEF undergoing PCI, machine learning models based on routinely available pre- and intra-procedural data enabled clinically meaningful post-PCI prognostic risk stratification of both 1-year adverse clinical outcomes and post-procedural LVEF improvement. The primary clinical model identified both high-risk and low-risk post-PCI phenotypes, whereas the LVEF improvement model provided complementary information regarding the likelihood of ventricular functional improvement. Together, these models provide a framework for individualized estimation of clinical and functional trajectories after PCI and may support prognostic assessment, follow-up planning, and patient counselling in this high-risk population. External validation and prospective evaluation are needed before clinical implementation.

## Supplementary Material

ztag156_Supplementary_Data

## Data Availability

The data used in this study are not publicly available due to institutional and privacy restrictions. The analytical code and trained models are not publicly available; however, methodological details are fully described in the manuscript to support reproducibility.
