# Supplementary material for: Machine learning-based prediction of clinical outcomes and left ventricular ejection fraction improvement after percutaneous coronary intervention in patients with reduced ejection fraction
Source: Eur Heart J Digit Health. 2026 Oct 27;7(9):ztag156. doi: 10.1093/ehjdh/ztag156 (PMC13711680; doi:10.1093/ehjdh/ztag156)
Supplement: Supplementary file 1 [file ztag156_supplementary_data.docx]

# Supplementary

## Table S1. Predictors included in model development

| **Category** | **Predictor** |
| --- | --- |
| Demographics | Age (years) |
|  | Female sex |
| Clinical history and comorbidities | Diabetes mellitus |
|  | Hypertension |
|  | Smoking history |
|  | Prior myocardial infarction |
|  | Chronic obstructive pulmonary disease |
|  | Peripheral vascular disease |
|  | Atrial fibrillation |
|  | Malignancy |
|  | Stroke |
|  | Dementia |
|  | Congestive heart failure |
| Laboratory variables | Hemoglobin (g/dL) |
|  | Platelet count (×10⁹/L) |
|  | Fibrinogen (mg/dL) |
|  | Total cholesterol (mg/dL) |
|  | High-density lipoprotein cholesterol (HDL) (mg/dL) |
|  | Low-density lipoprotein cholesterol (LDL) (mg/dL) |
|  | Triglycerides (mg/dL) |
|  | Uric acid (mg/dL) |
|  | White blood cell count (×10⁹/L) |
|  | Bilirubin (mg/dL) |
|  | Aspartate aminotransferase (AST) (U/L) |
|  | Alanine aminotransferase (ALT) (U/L) |
|  | Estimated glomerular filtration rate (MDRD) (mL/min/1.73 m²) |
| Echocardiographic parameters | Left ventricular ejection fraction (%) |
|  | Left ventricular end-diastolic diameter (mm) |
|  | Left ventricular end-systolic diameter (mm) |
|  | Interventricular septum thickness (mm) |
|  | Left ventricular posterior wall thickness (mm) |
|  | Left ventricular dysfunction (≥ mild) |
|  | Aortic stenosis grade (ordinal scale 0-6) |
|  | Aortic regurgitation grade (ordinal scale 0-6) |
|  | Mitral regurgitation grade (ordinal scale 0-6) |
|  | Tricuspid regurgitation grade (ordinal scale 0-6) |
|  | Prosthetic aortic valve |
|  | Prosthetic mitral valve |
|  | Inferior vena cava abnormality (binary) |
| Procedural and angiographic data | PCI count (including index procedure) |
|  | PCI performed for myocardial infarction |
|  | PCI performed for acute coronary syndrome |
|  | Cardiogenic shock at presentation |
|  | PCI completeness index >0.5 |
|  | Proximal major vessel treated |
|  | Graft treated |
|  | Circumflex artery treated |
|  | Left anterior descending artery treated |
|  | Left main artery treated |
|  | Right coronary artery treated |
| Prior cardiac intervention | Prior coronary artery bypass grafting (CABG) |
|  | Prior transcatheter aortic valve replacement (TAVR) |
|  | Prior PCI for ST-elevation myocardial infarction (STEMI) |

Predictors included demographic characteristics, clinical history and comorbidities, laboratory measurements, echocardiographic parameters, and procedural and angiographic variables available before or during the index PCI. PCI completeness was calculated as the ratio of coronary territories treated during the index procedure to the number of diseased vessels and categorized as higher (>0.5) versus lower (≤0.5). Continuous variables are presented with their measurement units where applicable. Valvular disease grades were included as ordinal scales in the models.

## Table S2. TRIPOD+AI checklist

| **Section** | **Item** | **Description** | **Where reported** |
| --- | --- | --- | --- |
| Title | 1 | Title identifies study as prediction model | Title |
| Abstract | 2 | Abstract summarizes objectives, methods, results | Abstract |
| Introduction | 3a | Background and rationale | Introduction |
|  | 3b | Intended use and target population | Introduction |
|  | 3c | Health inequalities considerations | Not specifically addressed |
|  | 4 | Study objectives | Introduction |
| Methods | 5a | Study design / data source | Methods - Study design |
|  | 5b | Study dates | Methods - Study design |
|  | 6a | Setting | Methods - Study design |
|  | 6b | Eligibility criteria | Methods - Study population |
|  | 6c | Treatment details | Methods - Study population |
|  | 7 | Data preprocessing | Methods - Model development |
|  | 8a | Outcome definition and timing | Methods - Outcome definitions |
|  | 8b | Outcome assessment | Methods - Outcome definitions |
|  | 8c | Blinding of outcome assessment | Not applicable |
|  | 9a | Predictor selection | Methods - Model development |
|  | 9b | Predictor definitions and measurement details | Methods - Model development; Supplementary Table S1 |
|  | 9c | Blinding of predictors | Not applicable |
|  | 10 | Sample size justification | Methods - Study population |
|  | 11 | Handling of missing data | Methods - Model development |
|  | 12a | Data splitting | Methods - Model development |
|  | 12b | Predictor handling | Methods - Model development |
|  | 12c | Model specification and tuning | Methods - Model development |
|  | 12d | Handling of heterogeneity | Not applicable |
|  | 12e | Performance measures | Methods - Model evaluation |
|  | 12f | Model updating | Not performed |
|  | 12g | Prediction generation | Methods - Model evaluation |
|  | 13 | Handling of class imbalance | Methods - Model development |
|  | 14 | Fairness considerations | Not specifically addressed |
|  | 15 | Model output format | Methods - Model evaluation |
|  | 16 | Development vs evaluation separation | Methods - Model development; Results - Table 1 |
|  | 17 | Ethics approval | Methods – Consent |
| Open science | 18a | Funding | Funding statement |
|  | 18b | Conflicts of interest | Disclosures |
|  | 18c | Protocol availability | No protocol was prepared |
|  | 18d | Registration | Not registered |
|  | 18e | Data availability | Data availability |
|  | 18f | Code availability | Data availability |
| Patient & public involvement | 19 | Patient & public involvement | No patient or public involvement |
| Results | 20a | Participant flow | Results - Study cohort; Figure 1 |
|  | 20b | Participant characteristics | Results - Table 1 |
|  | 20c | Comparison between datasets | Results - Table 1 |
|  | 21 | Sample sizes and outcome counts | Results - Study cohort; Figure 1 |
|  | 22 | Model details / availability | Methods - Model development; Data availability |
|  | 23a | Performance metrics | Results - Prediction of clinical outcomes; Prediction of LVEF improvement; Model performance in patients with baseline LVEF ≤40%; Tables 2-3; Figure 4; Supplementary Figure S1 |
|  | 23b | Heterogeneity in model performance across clusters | Not applicable |
|  | 24 | Model updating | Not performed |
| Discussion | 25 | Interpretation of results | Discussion |
|  | 26 | Limitations | Discussion |
|  | 27a | Handling of unavailable or poor-quality input data during implementation | Discussion |
|  | 27b | User interaction and expertise required to use the model | Discussion |
|  | 27c | Future work | Discussion |

## Table S3. Baseline characteristics of the exploratory favorable predicted outcome subgroup and remaining common test cases

| **Characteristic** | **Favorable predicted subgroup (N=23)** | **Remaining common test cases (N=257)** |
| --- | --- | --- |
| Diabetes mellitus | 4 (17.4%) | 161 (62.6%) |
| Left ventricular ejection fraction (%) | 30.0 [30.0, 36.0] | 35.0 [30.0, 40.0] |
| Estimated glomerular filtration rate (MDRD; mL/min/1.73 m²) | 83.2 [70.1, 92.0] | 71.6 [50.6, 92.1] |
| Uric acid (mg/dL) | 5.8 [5.3, 6.1] | 7.4 [6.1, 9.2] |
| Hemoglobin (g/dL) | 12.6 [11.8, 13.4] | 11.5 [9.5, 13.0] |
| Prior myocardial infarction | 1 (4.3%) | 111 (43.2%) |
| Age (years) | 64.0 [62.0, 74.0] | 68.0 [60.0, 74.0] |
| Left ventricular dysfunction (≥ mild) | 9 (39.1%) | 168 (65.4%) |
| Left ventricular end-diastolic diameter (mm) | 45.0 [44.0, 46.0] | 53.0 [49.0, 57.5] |

Values are presented as median [interquartile range] for continuous variables and n (%) for categorical variables. The favorable predicted subgroup was defined as cases with both low predicted MACE risk (bottom 25% of predictions) and high predicted probability of LVEF improvement (top 25% of predictions), compared with the remaining common test cases in the held-out test dataset. MDRD = Modification of Diet in Renal Disease.

## Table S4. Discharge cardiovascular and heart failure–related pharmacotherapy in the study cohort

| **Medication** | **Overall cohort** |
| --- | --- |
| Aspirin | 2259 (87.6%) |
| P2Y12 inhibitor / other antiplatelet therapy | 2466 (95.6%) |
| Oral anticoagulation | 436 (16.9%) |
| ACE inhibitor / angiotensin receptor blocker (ACEi/ARB) | 2076 (80.5%) |
| Beta-blocker | 2239 (86.8%) |
| Statin | 2441 (94.6%) |
| Diuretic | 1595 (61.8%) |
| Angiotensin receptor–neprilysin inhibitor (ARNI) | 116 (4.5%) |
| Sodium-glucose cotransporter-2 inhibitor (SGLT2i) | 265 (10.3%) |

Values are presented as n (%) based on non-missing observations for each medication. Medications were defined according to discharge treatment recorded after the index PCI hospitalization among cases included in the master analytical dataset. P2Y12 inhibitor / other antiplatelet therapy was defined as the presence of clopidogrel, ticagrelor, prasugrel, or other non-aspirin antiplatelet treatment. ACEi = angiotensin-converting enzyme inhibitor; ARB = angiotensin receptor blocker; ARNI = angiotensin receptor–neprilysin inhibitor; SGLT2i = sodium-glucose cotransporter-2 inhibitor.

Figure S1. Calibration curves for the MACE and LVEF improvement models on the held-out test dataset

Calibration curves comparing predicted probabilities with observed outcome rates in the held-out test dataset for the MACE (A) and LVEF improvement (B) models. Predicted probabilities were calibrated using Platt scaling. The diagonal line represents perfect calibration.

**Figure S1 (Alt Text):**
Two-panel calibration plot comparing predicted probabilities with observed outcome rates for the major adverse cardiovascular events model (A) and left ventricular ejection fraction improvement model (B) in the test dataset, with calibrated prediction curves shown against a diagonal reference line indicating perfect calibration.
